# PReS-FINAL-2099: Assessing the standards of oral health in children and adolescents with juvenile idiopathic arthritis

**DOI:** 10.1186/1546-0096-11-S2-P111

**Published:** 2013-12-05

**Authors:** SJ Trehane, H Strike, A Ramanan

**Affiliations:** 1 Paediatric and Adolescent Rheumatology Service, Bristol Royal Hospital for Children, Bristol, UK; 2 University of Bristol Medical School, Bristol, UK

## Introduction

Research has shown that Juvenile Idiopathic Arthritis (JIA) has an adverse effect on oral health. Patients with JIA have poor oral hygiene and higher levels of dental decay compared to healthy individuals [1]. The effect of JIA on manual dexterity and temporomandibular joint involvement can affect the patient's ability to perform good oral hygiene. Recent research has shown that other rheumatic disorders have a similar pathogenesis to periodontal disease. Oral health education is not routinely provided in the management of JIA. Good oral health can be achieved by following oral hygiene guidance, utilising dental health services and accessing dental interventions. Dental interventions which significantly reduce dental carries include fluoride varnish and fissure sealant; they are available to all children on the NHS.

## Objectives

The aim of the project was to assess the standards of oral health and hygiene in patients with JIA. To assess whether oral health education should be incorporated into the management of JIA.

## Methods

Patients diagnosed with JIA (aged 18 and under) were asked to complete an oral health questionnaire in rheumatology outpatient clinics. 97 questionnaires were completed. Data was analysed using Excel.

## Results

The age range was 18 months to 17 years of age; the average age was 10.3 years. 39 children have had fillings and 15 children have had teeth removed due to dental decay. A large proportion of the group were not following the recommended oral hygiene guidance. Uptake of dental interventions was low; only 14 children had fluoride varnish applied to their teeth and only 13 children had fissure sealants. (Table 1)

**Table 1:** Results from oral health questionnaire

*N 97*	Registered at dentist	Fillings	Teeth removed (decay)	Restricted mouth opening	Brushed teeth twice daily	Dental mouth-rinse	Electric tooth-brush	Sugared drink to bed	Sealants	Fluoride varnish
Y	95	39	15	12	70	26	34	14	13	14

N	2	58	82	85	27	71	63	83	84	83

DN	0	0	0	0	0	0	0	0	3	6

## Conclusion

The results show that a large proportion of the group had experienced dental decay and were not following the recommended oral health guidance. There was a low uptake of fluoride varnish and fissure sealants. The results emphasise that oral health education should be included in the management of JIA. Oral health education could be provided in multidisciplinary consultations and through the use of education booklets. Education should include oral hygiene advice and raise awareness about access to fluoride varnish and fissure sealants.

## Disclosure of interest

None declared.
